# Differential Effects of 3rd Trimester-Equivalent Binge Ethanol and Tobacco-Specific Nitrosamine Ketone Exposures on Brain Insulin Signaling in Adolescence

**Published:** 2016-02-26

**Authors:** Tomas Andreani, Ming Tong, Fusun Gundogan, Elizabeth Silbermann, Suzanne M. de la Monte

**Affiliations:** 1 Department of Medicine, Division of Gastroenterology, and the Liver Research Center Rhode Island Hospital, Providence, RI, USA; 2 Department of Pathology, Women and Infants Hospital of Rhode Island, Providence, RI, USA; 3 Departments of Pathology and Neurology, and the Division of Neuropathology, Rhode Island Hospital, Providence, RI, USA; 4 Warren Alpert Medical School of Brown University, Providence, RI, USA

**Keywords:** Tobacco, Nitrosamines, Fetal alcohol spectrum disorder, Multiplex ELISA

## Abstract

**Background:**

Fetal alcohol spectrum disorder (FASD) is associated with impairments in insulin and insulin-like growth factor (IGF) signaling through Akt pathways and altered expression of neuro-glial proteins needed for structural and functional integrity of the brain. However, alcohol abuse correlates with smoking, and tobacco smoke contains 4-(methylnitrosamino)-1-(3-pyridyl)-1-butanone (NNK), which like other nitrosamines, impairs insulin and IGF signaling.

**Hypothesis:**

NNK exposure can serve as a co-factor in mediating long-term neuro-developmental abnormalities associated with FASD.

**Design:**

Long Evans rat pups were IP administered ethanol (2 g/kg) on postnatal days (P) 2, 4, 6 and/or NNK (2 mg/kg) on P3, P5, and P7, simulating third trimester human exposures. Temporal lobes from P30 rats (young adolescent) were used to measure signaling through the insulin/IGF-1/Akt pathways by multiplex ELISAs, and expression of neuroglial proteins by duplex ELISAs.

**Results:**

Ethanol, NNK, and ethanol + NNK exposures significantly inhibited insulin receptor tyrosine phosphorylation, and IRS-1 and myelin-associated glycoprotein expression. However, the major long-term adverse effects on Akt pathway downstream signaling and its targeted proteins including choline acetyltransferase, Tau, pTau, ubiquitin, and aspartate-β-hydroxylase were due to NNK rather than ethanol.

**Conclusion:**

Alcohol and tobacco exposures can both contribute to long-term brain abnormalities currently regarded fetal ethanol effects. However, the findings suggest that many of the adverse effects on brain function are attributable to smoking, including impairments in signaling through survival and metabolic pathways, and altered expression of genes that regulate myelin synthesis, maturation and integrity and synaptic plasticity. Therefore, public health measures should address both substances of abuse to prevent “FASD”.

## Introduction

Chronic alcohol abuse causes cognitive impairment and neurodegeneration in which corticolimbic structures, the cerebellum, and white matter are major targets [1]. Previous human and experimental animal studies demonstrated roles for brain insulin and insulin-like growth factor type 1 (IGF-1) resistance, together with increased oxidative stress as mediators of neurodegeneration [2–7]. Alcohol-related impairments in brain insulin and IGF-1 signaling are associated with reduced insulin and IGF-1 receptor tyrosine phosphorylation, decreased signaling through insulin receptor substrate proteins, phospho-inositol-3-kinase (PI3K), and Akt, increased activation of glycogen synthase kinase 3β (GSK-3β), and attendant reductions inneuronal cholinergic function [7–10]. Since insulin signaling through PI3K-Akt mediates cell survival, metabolism, and neuronal plasticity [11], addition consequences of insulin resistance include oxidative stress, DNA damage, loss of neuronal plasticity and repair, and deficits in energy balance. Oxidative stress and DNA damage contribute to ethanol-associated mitochondrial dysfunction, which further increases stress, neuro-inflammation, and insulin resistance [12–17].

Variability in the nature and severity of alcohol-related neurodegeneration suggests that co-factors may be critical to disease pathogenesis. In this regard, it is noteworthy that a very high percentage of heavy drinkers (up to 80%) also abuse tobacco products, typically by smoking cigarettes [18]. Although the overwhelming interest in studying adverse effects of alcohol-tobacco dual exposures has focused on carcinogenesis [19–22], particularly in relation to the tobacco-specific nitrosamine, 4-(methylnitrosamino)-1-(3-pyridyl)-1-butanone (NNK) and its metabolites [20.^23^], previous studies demonstrated that limited, sub-mutagenic exposures to other nitrosamines, i.e. streptozotocinor N-nitrosodiethylamine (NDEA), cause brain insulin resistance, increased DNA damage, lipid peroxidation, mitochondrial dysfunction, ER stress, and impaired signaling through PI3K-Akt (24–26) and can exacerbate effects of ethanol [27]. The present study tests the hypothesis that sub-mutagenic exposures to NNK are sufficient to cause neurodegeneration and possibly exacerbate the adverse effects of alcohol with respect to brain insulin/IGF resistance, oxidative stress, neuroglial gene expression, and myelin maintenance. NNK rather than tobacco smoke effects were studied because smoking could confound the results by causing pulmonary disease [28,29].

## Methods

### In vivo Model

Long Evans rat pups were divided into four groups and administered 50 μl IP injections of: saline vehicle as control; pharmaceutical grade ethanol (2g/kg in saline); NNK (2 mg/kg in saline); and ethanol + NNK. Ethanol treatments (binge) were administered on postnatal days (P) 2, 4, 6, and 8 [30–32], and NNK was administered on P3, P5, P7, and P9. These models simulated 3^rd^ trimester-equivalent human pregnancy exposures to alcohol and/or tobacco toxins. The rats were sacrificed at six weeks of age to examine long-term effects on temporal lobe insulin/IGF-1 signaling through Akt growth and metabolic pathways during late adolescence. All experiments were performed in accordance with protocols approved by Institutional Animal Care and Use Committee at the Lifespan-Rhode Island Hospital, and they conformed to guidelines established by the National Institutes of Health.

### Preparation of Protein Homogenates

Protein homogenates were prepared in lysis buffer containing 50 mM Tris (pH 7.5), 150 mM NaCl, 5 mM EDTA (pH 8.0), 50 mM NaF, 0.1% Triton X-100, and protease (1mM PMSF, 0.1 mM TPCK, 1 mg/ml aprotinin, 1 mg/ml pepstatin A, 0.5 mg/ml leupeptin, 1 mM NaF, 1 mM Na_4_P_2_O_7_) and phosphatase (2 mM Na_3_VO_4_) inhibitors [8]. To accomplish this, snap-frozen tissue samples (50 mg) were homogenized for 2 minutes in a TissueLyser II (Qiagen, Germantown, MD) using 5 mm stainless steel beads. Protein homogenates were centrifuged at 14000xg for 10 min at 4°C and supernatant fraction protein concentrations were measured by the bicinchoninic acid (BCA) assay.

### Duplex Enzyme-linked Immunosorbent Assays (ELISAs)

Direct binding duplex ELISAs measured immunoreactivity with results normalized to large acidic ribosomal protein (RPLPO) [33]. Immunoreactivity to target proteins was detected with horseradish peroxidase-conjugated secondary antibody and Amplex UltraRed soluble fluorophore (Invitrogen, Carlsbad, CA). RPLPO antibody (Proteintech Group Inc, Chicago, IL) was biotinylated, and its immunoreactivity was detected with streptavidin-conjugated alkaline phosphatase and the 4-Methylumbelliferyl phosphate (4-MUP) substrate. Fluorescence intensities (Amplex Red: Ex 565 nm/Em 595 nm; 4-MUP: Ex360/Em450) was measured in a SpectraMax M5 (Molecular Devices, Sunnyvale, CA). Antibody omission controls were included. The calculated target protein/RPLPO ratios were used for inter-group comparisons.

### Bead-based Multiplex ELISAs

We used bead-based multiplex ELISAs to measure immunoreactivity to the insulin receptor (IR), IGF-1 receptor (IGF-1R), IRS-1, Akt, proline-rich Akt substrate of 40 kDa (PRAS40), ribosomal protein S6 kinase (p70S6K), and glycogen synthase kinase 3β (GSK-3β), and ^pYpY1162/1163^-IR, ^pYpY1135/1136^-IGF-1R, ^pS312^-IRS-1, ^pS473^-Akt, ^pT246^-PRAS40, ^pTpS421/424^-p70S6K, and ^pS9^-GSK-3β (Invitrogen, Carlsbad, CA). Samples (100 μg protein) were incubated with the beads, and captured antigens were detected with secondary antibodies and phycoerythrin-conjugated anti-rabbit IgG [33]. Plates were read in a MAGPIX (Bio-Rad, Hercules, CA).

### Statistics

For competitive, duplex, and multiplex ELISAS, each experimental group included 8–10 rats. Inter-group comparisons were made using two-way analysis of variance (ANOVA) with Tukey post hoc tests (GraphPad Prism 6, San Diego, CA). F-ratios and P-values are tabulated. Significant post hoc test differences and trends (*0.05 < P < 0.10*) are shown in the graphs.

## Materials

Pharmaceutical grade ethanol was used in the in vivo experiments. The A85G6 and A85E6 monoclonal antibodies to ASPH were generated to human recombinant protein [34] and purified over Protein G columns (Healthcare, Piscataway, NJ). Otherwise, antibodies used for duplex ELISAs were purchased from Abcam (Cambridge, MA). RPLPO) antibody was from the Proteintech Group Inc (Chicago, IL). ELISA MaxiSorp 96-well plates were purchased from Nunc (Rochester, NY). Horseradish peroxidase (HRP)-conjugated secondary antibody, Amplex Red soluble fluorophore, and the Akt Pathway Total and Phospho panels were purchased from Invitrogen (Carlsbad, CA). HRP-labeled polymer conjugated secondary antibody was purchased from Dako Corp (Carpinteria, CA). The SpectraMax M5 microplate reader was purchased from Molecular Devices Corp. (Sunnyvale, CA). BCA reagents were from Pierce Chemical Corp. (Rockford, IL). All other fine chemicals, including NNK were purchased from CalBiochem (Carlsbad, CA), Pierce (Rockford, IL), or Sigma (St. Louis, MO).

## Results

### Ethanol and NNK Effects on Mediators of Insulin and IGF-1 Signaling

**Figure 1:**
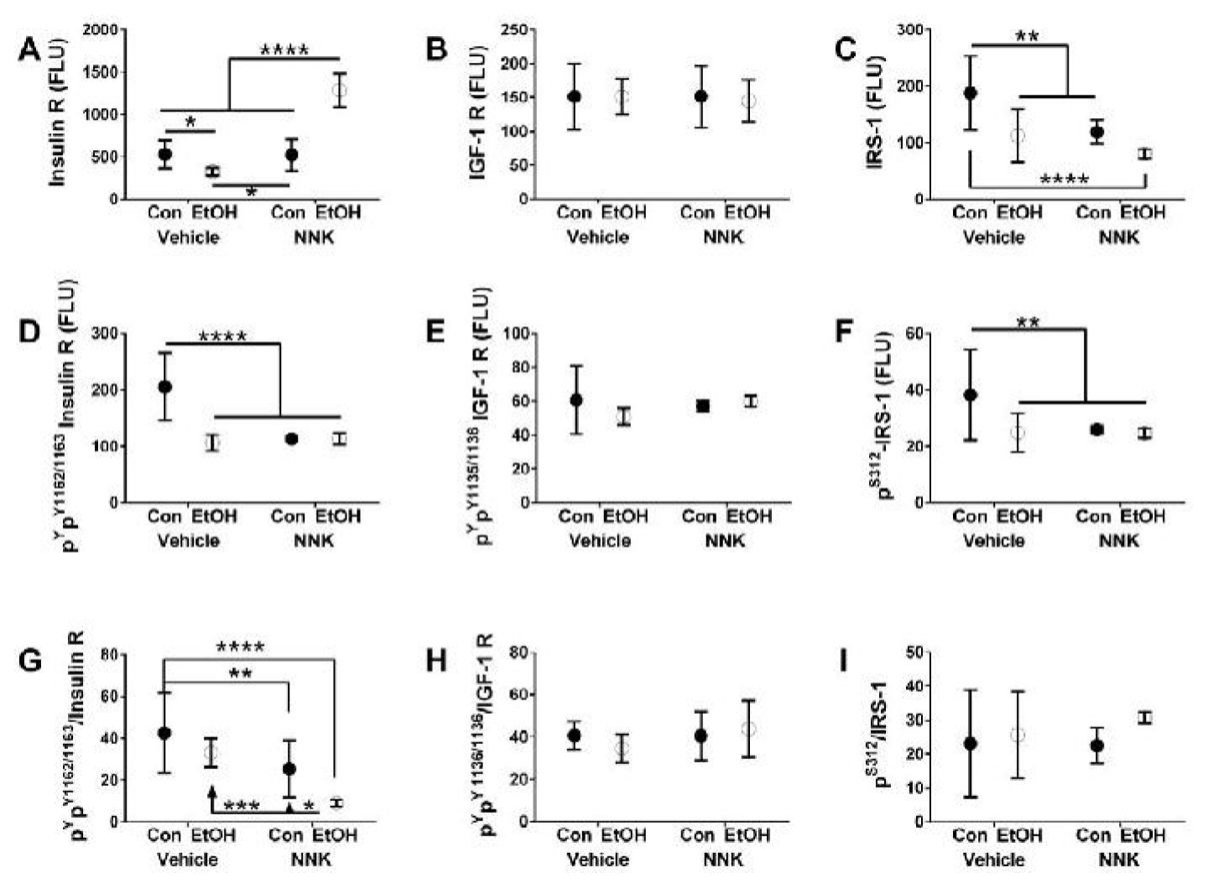
Ethanol, NNK and ethanol + NNK effects on temporal lobe expression of upstream regulators of insulin/IGF signaling. Bead-based multiplex ELISAs were used to measure immunoreactivity to (A) insulin R, (B) IGF-1R, (C) IRS-1, (D) ^pYpY1162/1163^-Insulin R, (E) ^pYpY1135/1136^-IGF-1R, and (F) ^pS312^-IRS-1, The calculated mean ratios of (G) ^pYpY1162/1163^-/total Insulin R, (H) ^pYpY1135/1136^-/total IGF-1R, (I) ^pS312^-/total IRS-1 reflect relative levels of phosphorylation. Data was analyzed by two-way ANOVA (Table 1). Graphs depict levels of immunoreactivity (Fluorescent light units-FLU: mean ± S.D). Significant differences obtained by post-hoc Tukey multiple comparison tests are depicted in the graphs (*P < 0.05; ** *P < 0.01*; ***P < 0.001; ****P < 0.0001).

**Figure 2:**
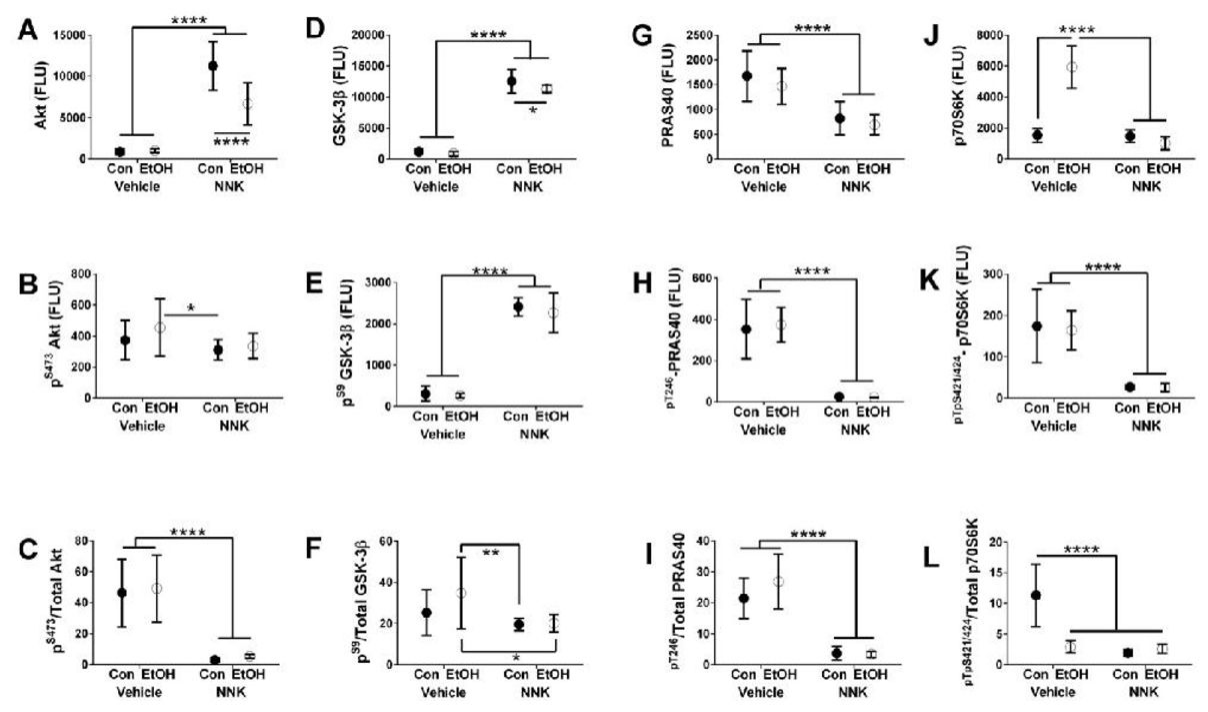
Ethanol, NNK and ethanol + NNK effects on insulin/IGF-Akt pathway activation. Temporal lobe protein homogenates were used in bead-based multiplex ELISAs to measure immunoreactivity to (A) Akt, (D), GSK-3β, (G) PRAS40, (J) p70S6K,(B) ^pS473^ AKT, (E) ^pS9^-GSK-3β, (H) ^pT246^-PRAS40, and (K) ^pTpS421/424^-p70S6K. The calculated mean ratios of (C) ^pS473^-total AKT, (F) ^pS9^-/total GSK-3β, (I) ^pT246^-/total PRAS40, and (L) ^pTpS421/424^-/total p70S6K) reflect relative levels of phosphorylation. Data were analyzed by two-way ANOVA (Table 1). Graphs depict levels of immunoreactivity (Fluorescent light units-FLU: mean ± S.D.). Significant differences obtained by post-hoc Tukey multiple comparison tests are depicted in the graphs (*P < 0.05; **P < 0.01; ***P < 0.001; ****P < 0.0001).

Total and phosphorylated levels of insulin receptor (Insulin R), IGF-1 receptor (IGF-1R), IRS-1, Akt, GSK-3β, and p70S6K were measured using bead-based multiplex ELISAs. Levels of relative phosphorylation of the Insulin R, IGF-1R, IRS-1, Akt, GSK-3β, and p70S6K were calculated from the ratios of phosphorylated/total (p/T) proteins. Data were analyzed using two-way ANOVA (Table 1) and post hoc repeated measures Tukey tests (Figures 1–2).

### Signaling Proteins

The two-way ANOVA tests demonstrated that ethanol had significant effects on insulin R, IRS-1, Akt, and GSK-3β expression; NNK had significant effects on all signaling proteins except IGF-1R; and ethanol × NNK interactive effects were significant for insulin R and Akt (Table 1). Graphs, together with post hoc Tukey repeated measures tests demonstrated that ethanol significantly reduced the mean expression levels of Insulin R (Figure 1A) and IRS-1(Figure 1C), but increased p70S6K (Figure 2J) relative to control. The effects of NNK and ethanol + NNK were largely similar in that both significantly reduced IRS-1 expression relative to control (Figure 1C), and increased Akt (Figure 2A) and GSK-3β (Figure 2D) but decreased PRAS40 (Figure 2G) relative to both control and ethanol groups. NNK and ethanol + NNK effects were distinguished by the significantly higher levels of insulin R expression in the ethanol + NNK group relative to the other three groups (Figure 1A), and lower levels of Akt (Figure 2A) and GSK-3β (Figure 2D) in samples from ethanol + NNK treated relative to NNK only. There were no significant inter-group differences with respect to IGF-1R expression (Figure 1B).

### Phosphorylated Signaling Proteins

The two-way ANOVA tests demonstrated significant effects of ethanol on of ^pYpY1162/1163^-Insulin R and ^pS312^-IRS-1, significant effects of NNK on ^pYpY1162/1163^-Insulin R, ^pS473^-Akt, ^pS9^-GSK-3β, ^pTpS421/424^-p70S6K, and ^pT246^-PRAS40, and trend effects (*0.05 < P < 0.10*) on ^pS312^-IRS-1. Significant ethanol × NNK interactive effects occurred with respect to ^pYpY1162/1163^-Insulin R, while trend effects were observed for^pS312^-IRS-1 and ^pYpY1135/1136^-IGF-1R. Post hoc tests to examine specific intergroup differences beyond overall effects demonstrated significantly reduced levels of ^pYpY1162/1163^-Insulin R (Figure 1D) and ^pS312^-IRS-1 (Figure 1F) in the ethanol, NNK and ethanol + NNK groups relative to control. In addition, ^pS9^-GSK-3β (Figure 2F), and ^pS473^-Akt in the ethanol, NNK and ethanol + NNK groups relative to control (Figures 2A and 2D). In addition, ^pS9^-GSK-3β was significantly elevated and ^pT246^-PRAS40 (Figure 2G) and ^pTpS421/424^-p70S6K (Figure 2H) were sharply and significantly reduced in the NNK and ethanol + NNK groups relative to control and ethanol treatment. Additive or interactive effects of ethanol and NNK were not observed and none of the treatments significantly altered expression of ^pYpY1135/1136^-IGF-1R (Figure 1E).

### Relative Phosphorylation of Signaling Proteins

With regard to the relative levels of phosphorylation (p/T), ethanol had significant effects on the ^pYpY1162/1163^-Insulin R/total Insulin R and ^pTpS421/424^-p70S6K/total p70S6K. NNK had significant effects on ^pYpY1162/1163^-Insulin R/total Insulin R, ^pS473^-Akt/total Akt, ^pS9^-GSK-3β/total GSK-3β, ^pTpS421/424^-p70S6K/total p70S6K, and ^pT246^-PRAS40/total PRAS40. Ethanol × NNK interactive effects were significant only with respect to ^pTpS421/424^-p70S6K/total p70S6K. The graphs and post hoc tests demonstrated progressive declines in the mean levels of ^pYpY1162/1163^-Insulin R/total Insulin R from control to ethanol, then NNK, and finally ethanol + NNK (Figure 1G). In addition, the mean levels of ^pS473^-Akt/total Akt (Figure 2C and ^pT246^-PRAS40/total PRAS40 (Figure 2I) were significantly lower in the NNK and ethanol + NNK groups relative to control and ethanol, and the levels of ^pTpS421/424^-p70S6K/total p70S6K (Figure 2L) were significantly reduced in all three experimental groups relative to control. The slightly increased mean level of ^pS9^-GSK-3β/total GSK-3β in the ethanol group rendered the differences from NNK and ethanol + NNK statistically significant (Figure 2F). Finally, there were no significant treatment effects on the levels of ^pYpY1135/1136^-IGF-1R/total IGF-1R (Figure 1H) or ^pS312^-IRS-1/total IRS-1 (Figure 1I). Overall, most of the inhibitory effects on both proximal and distal components of the insulin/IGF signaling network were driven by NNK, with or without co-exposure to ethanol. The main inhibitory effects of ethanol were on insulin receptor expression and tyrosine phosphorylation, IRS-1 expression, and relative phosphorylation of p70S6K.

### Ethanol and NNK Effects on Neuronal, Glial, and Stress Proteins

To determine the consequences of impaired insulin/IGF-1 signaling altered expression of structural and functional neuroglial proteins and increased oxidative stress, we measured immunoreactivity to Hu (neuronal), myelin-associated glycoprotein-1 (MAG-1; oligodendroglia), glial fibrillary acidic protein (GFAP; astrocytes), choline acetyl transferease (ChAT), acetylcholinesterase (AChE), glyceraldehyde-3-phosphate dehydrogenase (GAPDH), tau, phospho-tau, ubiquitin, 4-hydroxy-2-nonenal (HNE), and aspartyl-asparaginyl-β-hydroxylase (ASPH). ASPH-A85G6 detects the C-terminal catalytic domain that confers cell motility [35–39], while ASPH-A85E6 binds to the N-terminal region corresponding to Humbug, which regulates calcium flux from the ER and cell adhesion [40]. Duplex ELISA results were normalized to RPLPO as a reference for protein loading [33].

**Figure 3:**
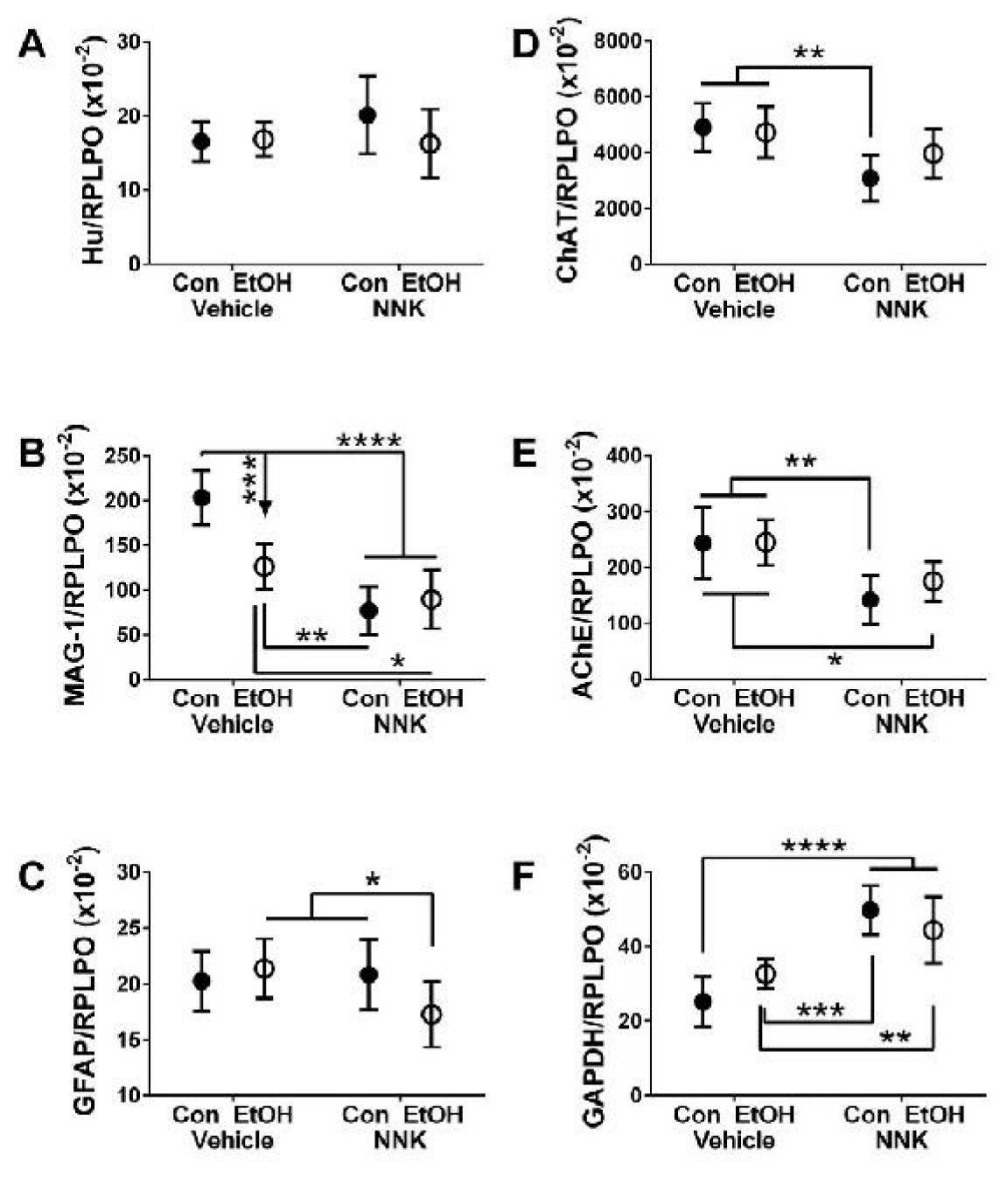
Ethanol, NNK and ethanol + NNK effects on neuronal and glial protein expression. Duplex ELISAs were used to measure Immunoreactivity to (A) Hu, (B) myelin-associated glycoprotein 1 (MAG-1), (C) glial fibrillary acidic protein (GFAP), (D) choline acetyltransferase (ChAT), (E) acetylcholinesterase (AChE), and (F) glyceraldehyde-3-phosphate dehydrogenase (GAPDH) with results normalized to RPLPO (control). Data were analyzed by two-way ANOVA (Table 2). Post hoc Tukey repeated measures tests detected significant inter-group differences as shown in the panels: *P < 0.05; **P < 0.01; ***P < 0.001; ****P < 0.0001.

**Figure 4:**
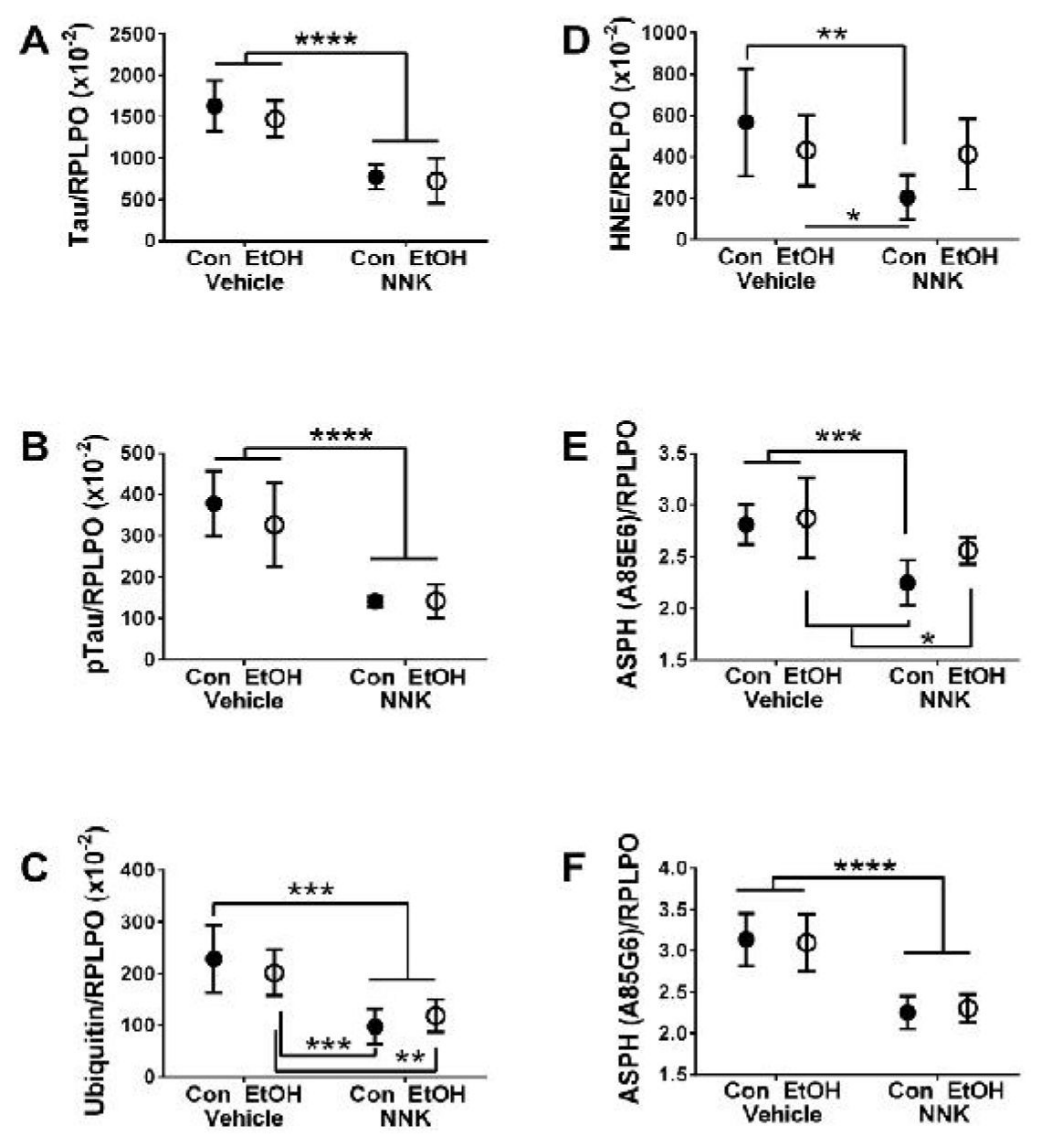
Long-term effects of developmental ethanol, NNK and ethanol + NNK exposures on neuronal and stress proteins. Temporal lobe protein homogenates were used to measure (A) Tau, (B) pTau, (C) ubiquitin, (D) 4-hydroxy-2-nonenal (HNE), (E) aspartate-β-hydroxylase (Humbug ASPHA85E6), and (F) ASPH-A85G6 (catalytic domain) immunoreactivity by duplex ELISAs with results was normalized to RPLPO. Inter-group comparisons were made by two-way ANOVA (Table 2). Post hoc Tukey repeated measures tests detected significant inter-group differences as shown in the panels: *P < 0.05; **P < 0.01; ***P < 0.001; ****P < 0.0001.

Two-way ANOVA tests revealed significant ethanol effects on the expression of MAG-1 and a trend effect on ASPH-A85E6, and significant NNK effects on the expression of all proteins measured except Hu and GFAP, and the calculate pTau/Tau ratio (Table 2). Significant ethanol × NNK interactive effects were detected for MAG-1, GAPDH, and HNE, while a trend effect was detected for GFAP. The graphs in Figures 3 and 4 illustrate specific effects of the various exposures on protein expression. Hu was similarly expressed in all groups (Figure 3A), whereas MAG-1 was significantly reduced in all experimental groups relative to control (Figure 3B). Furthermore, MAG-1 expression, a marker of mature oligodendrocyte function, was significantly lower in the NNK and ethanol + NNK temporal lobe samples than in the ethanol-only group. GFAP, which reflects astrocyte function, was significantly reduced in the ethanol + NNK group relative to the ethanol- and NNK-only groups (Figure 3C).

ChAT (Figure 3D) and AChE (Figure 3E), which regulate cholinergic homeostasis, were similarly reduced by NNK and ethanol + NNK exposures, rendering the differences from control statistically significant. GAPDH expression was significantly increased in both NNK and ethanol + NNK groups relative to control and ethanol-only treatment (Figure 3F). The effects of ethanol, NNK, and ethanol + NNK exposures on Tau (Figure 4A), pTau (Figure 4B), ubiquitin (Figure 4C), ASPH-A85E6 (Figure 4E) and ASPH-A85G6 (Figure 4F) expression were thematically similar in that ethanol had minimal effect relative to control, while NNK and ethanol + NNK reduced protein expression relative to both control and/or ethanol-only treatment. The calculated pTau/Tau ratios did not differ among the groups (Table 2) because the pTau levels were mainly driven by Tau protein expression rather than differential alterations in pTau. Regarding both HNE (Figure 4D) and ASPHA85E6 (Figure 4E), the inhibitory effects of ethanol + NNK were less than for NNK only, rendering the differences from control not statistically significant.

## Discussion

### Early Postnatal Ethanol and NNK Exposure Model

This study examines long-term effects of early postnatal ethanol and NNK exposures on insulin and IGF-1 signaling through Akt pathways in adolescent rat temporal lobes. The experiment was designed to mimic binge drinking and smoking in the third trimester of human pregnancy. Our working hypothesis was that low-dose NNK exposures, which occur with first- or secondhand smoking, could mediate long-term impairments in brain insulin/IGF-1 signaling through Akt pathways, and thereby cause phenotypic effects that overlap with FASD.

Previous studies revealed that chronic ethanol exposures cause significant sustained impairments in insulin signaling in various organs, including brain, and in both humans and experimental animals [5,7,14,33,41–47]. Ethanol-mediated impairments in insulin/IGF-1 signaling occur through survival and metabolic pathways and are associated with increased GSK-3β activation, oxidative stress, and cell death [5,7,14,33,41–46]. Previous studies were directed toward cerebellar and frontal lobe pathology. The temporal lobe is yet another target of alcohol neurotoxicity, and of interest due to its role in learning and memory.

### Ethanol and NNK Effects on Temporal Lobe Insulin/IGF-1/IRS-1 Signaling

The major findings were that: 1) ethanol and NNK independently altered the expression of proteins and phospho-proteins that mediate upstream and downstream components of the insulin R/IRS-1/Akt pathway, but had no significant effect on IGF-1R signaling; and 2) NNK, with or without ethanol co-exposure, was the main driver of impaired signaling through Akt networks that support cell survival, plasticity, and metabolism. In essence, NNK’s effects were highly significant through most of the downstream steps; whereas ethanol’s adverse effects were more limited its upregulation of p70S6K and inhibition of its relative phosphorylation. The finding that both ethanol and NNK inhibited ^pYpY1162/1163^-Insulin R expression is evidence that either alcohol or tobacco smoke exposures early in development can lead to sustained impairment of insulin signaling in adolescent brains, corresponding with previously reported effects in experimental FASD [27,48,49]. This concept is reinforced by potentially additive effects of dual exposures in which the temporal lobe levels of ^pYpY1162/1163^-Insulin R were lowest among the groups, despite paradoxically increased insulin R expression. The absence of ethanol and NNK effects on IGF-1R and ^pYpY1135/1136^-IGF-1R is discordant with previous findings [27,48,49]; however, the differences could be structure-dependent since the previous work focused on the cerebellum rather than the temporal lobe.

The greater reduction in IRS-1 protein expression in the ethanol + NNK compared with either ethanol or NNK suggests that the adverse effects of the dual exposures were additive. However, the corresponding reductions in ^S312^-IRS-1 in all 3 experimental groups parallel declines in IRS-1 protein, and since S312 phosphorylation of IRS-1 is inhibitory, it is unlikely that the decreases in downstream Akt signaling were not due to disruption of IRS-1 phosphorylation, and instead were mediated by decreased levels of IRS-1 protein.

In contrast to previous work in which chronic prenatal or early postnatal binge ethanol exposures were shown to have striking inhibitory effects on Akt, GSK-3β, and PRAS40 phosphorylation in the cerebellum [27,48,49], no such responses to ethanol occurred in the temporal lobe. Instead, the main downstream effects of ethanol were to increase p70S6K protein while substantially inhibiting its relative levels of phosphorylation. p70S6K, which is downstream of Akt and connected through the mammalian target of rapamycin (mTOR) pathway, promotes protein synthesis. In the brain, mTOR/p70S6K mediates brain-derived neurotrophic factor-induced protein synthesis and neuroplasticity (50), and therefore ethanol inhibition of p70S6K activation in the temporal lobe could lead to sustained impairment of neuronal plasticity required for learning and memory.

NNK, with or without ethanol co-exposures, broadly inhibited Akt pathway signaling relative to control and/or ethanol exposure. With regard to Akt and GSK-3β, the NNK-associated increases in protein may have been compensatory. However, due to the absence of corresponding increases in protein phosphorylation, the relative levels of ^pS473^-Akt and ^S9^-GSK-3β were reduced. (Note that S9 phosphorylation of GSK-3β inhibits the kinase activity). In addition, NNK and ethanol + NNK significantly inhibited expression of PRAS40, ^pT246^-PRAS40, p70S6K, and ^pTpS421/424^-p70S6K, causing their relative levels to also be reduced. In essence, the net long-term effects of early postnatal NNK exposures were to inhibit virtually the entire insulin signaling pathway from receptor through downstream Akt networks that support neuronal survival, energy metabolism, protein synthesis, and plasticity. In these respects, early postnatal NNK effects on the temporal lobe mimic the longterm effects of binge ethanol exposures on the cerebellum [27,48]. Furthermore, the findings suggest that the impairments in signaling were mainly driven by NNK, since there were virtually no additive effects of the dual exposures.

### Differential effects of ethanol and NNK on neuronal and glial protein expression

Ethanol, NNK, and ethanol + NNK exposures all significantly reduced temporal lobe levels of MAG-1 expression relative to control, although the effects of NNK and ethanol + NNK were more pronounced than ethanol’s. MAG-1, a glycoprotein expressed in oligodendrocytes, is responsible for facilitating cell-cell interactions between neuronal and myelinating cells. Ethanol’s inhibitory effects on white matter development are well-established and have been linked to impairments in oligodendrocyte myelin-associated gene/protein expression [1]. The finding that developmental exposures to NNK can also reduce MAG-1 expression is novel and supports the hypothesis that alcohol and tobacco smoke exposures can both contribute to white matter hypotrophy and reduced myelination in adolescent brains. In contrast, there were no significant differences in the expression levels of Hu, a marker of neurons, or GFAP, the main intermediate filament protein of mature astrocytes, in the experimental groups relative to control. These findings highlight the selective targeting of oligodendrocytes by ethanol and NNK.

Acetylcholine, one of the major neurotransmitters utilized for neuronal plasticity in the brain, is regulated by ChAT for biosynthesis, and AChE for degradation. The absence of ethanol effects on ChAT and AChE is discordant with previous findings in studies of the cerebellum [5,8,14]. We speculate that rapidly proliferating, migrating and differentiating neurons in early postnatal cerebella are more vulnerable as targets of ethanol neurotoxicity than post-mitotic temporal lobe neurons. On the other hand, the findings that ChAT and/or AChE expression were reduced by developmental exposures to NNK suggest that postmitotic temporal lobe neurons are susceptible to the delayed neurotoxic effects of NNK. Reduced expression of ChAT correlates with impaired insulin signaling [8]. Inhibition of AChE expression can be mediated by oxidative stress [51–54], such as that caused by the impairments of insulin signaling through Akt with increased activation of GSK-3β, as occurred in brains from NNK-exposed rats. Inhibition of AChE can be sufficient to cause cytoskeletal collapse and neurodegeneration [55]. Together, these findings suggest that NNK and therefore tobacco smoke exposures in the early postnatal period (equivalent to 3^rd^ trimester of human pregnancy) can impair temporal lobe cholinergic function which is needed for neuronal plasticity, learning and memory.

Further studies showed that NNK and ethanol + NNK similarly reduced tau, p-tau, ubiquitin, ASPH-A85G6 and ASPH-A85E6 protein expression relative to control and ethanol exposures. These responses were driven by NNK since ethanol had no independent or additive effects. Tau is a major neuronal cytoskeletal protein whose phosphorylation state is critical for translocation from the perikarya into neurites for establishing and maintaining synaptic connections. Therefore, NNK’s Inhibition of Tau and p-Tau expression could reflect retraction or degeneration of axons, collapse of growth cone, and synaptic disconnection [56]. Since tau expression and phosphorylation are regulated by insulin and IGF-1 signaling through Akt and GSK-3β [57–59], it is not surprising that these proteins were significantly reduced by NNK exposures, given the prominent inhibition of insulin Rand Akt phosphorylation. The finding that the relative levels of pTau (pTau/Tau) were not significantly reduced vis-à-vis significant reductions Tau and pTau following NNK exposure indicates that the main effect of NNK was to inhibit Tau expression. The similarly reduced levels of pTau are best explained by the lower levels of protein rather than impaired signaling and kinase activation via GSK-3β. On the other hand, in ethanol-exposed temporal lobes, the relatively normal levels of tau and pTau could be explained by preservation of signaling through Akt and GSK-3β. The NNK associated reductions in ubiquitin could reflect deficits in the ubiquitin-proteasome system. A similar response occurs following chronic ethanol exposure [60,61]. Deficits in the ubiquitin-proteasome pathway could lead to increased oxidative and endoplasmic reticulum stress due to activation of the unfolded protein response [62,63].

For ASPH, we used the A85G6 monoclonal antibody that binds to the C-terminal region of ASPH which contains a catalytic domain, and A85E6, that binds to the N-terminal Humbug-homologous region of ASPH [34,40,64]. The catalytic domain of ASPH is required to promote cell motility [35–37,65,66] and neuronal plasticity [35–40,67]. Humbug regulates calcium sequestration in the ER (68). ASPH expression and function are regulated by insulin/IGF-1 signaling through IRS-1 and Akt [35,40,67]. Inhibition of ASPH perturbs cell motility and adhesion [36,39,69], and in the case of FASD, ethanol’s inhibitory effects on ASPH expression correlate with impairments in cerebellar neuronal migration and motor dysfunction [34,64]. The findings herein demonstrate that early post-natal exposures to NNK significantly inhibit temporal lobe expression of ASPH and Humbug, correlating with reduced activation of Akt. In contrast, ethanol had no significant effect on these proteins, corresponding with the preservation of signaling through Akt and GSK-3β in the temporal lobe.

In conclusion, ethanol and NNK exposures during early postnatal development impaired signaling through the insulin receptor and IRS-1. However, downstream signaling through Akt/GSK-3β was significantly compromised by NNK and not ethanol. Long-term adverse effects shared by ethanol and NNK exposures include inhibition of p70S6K phosphorylation and MAG-1 expression in the temporal lobe. In contrast, NNK exposures had broad sustained adverse effects associated with impairments in downstream signaling through the Akt pathway and its target proteins. It is noteworthy that these 3^rd^ trimester-equivalent NNK exposure effects are similar to those produced in the cerebellum and temporal lobe by chronic prenatal (1^st^ and 2^nd^ trimester) ethanol exposures [14], and in the cerebellum following postnatal binge (3^rd^ trimester) ethanol exposures [70]. The differential responses to ethanol and NNK highlight the concept that the developmental windows and targets of ethanol [71] versus NNK mediated impairments in brain function overlap but are not identical in that effects can vary based on timing (chronic versus binge), developmental age, and developmental stage of the targeted region of brain. These studies illustrate how alcohol and tobacco smoke exposures during development can both contribute to brain abnormalities currently designated as FASD.

## Figures and Tables

**Table 1:** Two-Way ANOVA summary of ethanol and NNK effects on Insulin/IGF-1/Akt signaling networks in the temporal lobe-multiplex ELISA results (Temporal lobe protein homogenates were used to measure total and phosphorylated (p) proteins in the insulin/IGF-1/IRS-1 Akt pathway by multiplex bead-based ELISAs. In addition, the ratios of phosphorylated/total (p/T) protein were calculated. Data were analyzed by Two-way ANOVA with the post-hoc Tukey test. Italicized values indicate statistical trends. Data are graphed in Figures 1–2).

Protein	Ethanol Effect		NNK Effect		Ethanol × NNK Effect	
	F-Ratio	P-Value	F-Ratio	P-Value	F-Ratio	P-Value
Insulin R	24.25	< 0.0001	71.18	< 0.0001	73.20	<0.0001
IGF-1 R	0.061	N.S.	0.048	N.S.	0.063	N.S.
IRS-1	14.92	0.0006	11.66	0.002	1.526	N.S.
Akt	10.26	0.0034	133.6	< 0.0001	11.28	0.0023
GSK-3β	4.410	0.0045	871.1	< 0.0001	1.377	N.S.
P70S6K	1.657	N.S.	38.95	< 0.0001	0.087	N.S.
PRAS40	1.657	N.S.	38.95	< 0.0001	0.087	N.S.
p-Insulin-R	20.00	0.0001	14.87	0.0006	20.33	0.0001
p-IGF-1R	0.789	N.S.	0.498	N.S.	2.842	0.100
p-IRS-1	5.440	0.027	*4.043*	*0.054*	*3.788*	*0.062*
p-Akt	1.449	N.S.	4.333	0.047	0.408	N.S.
p-GSK-3β	1.001	N.S.	435.2	< 0.0001	0.262	N.S.
p-p70S6K	0.098	N.S.	64.14	< 0.0001	0.061	N.S.
p-PRAS40	0.100	N.S.	134.7	< 0.0001	0.179	N.S.
p/T-Insulin R	8.789	0.006	22.70	< 0.0001	0.671	N.S.
p/T-IGF-1R	0.135	N.S.	1.661	N.S.	1.175	N.S.
p/T-IRS-1	2.118	N.S.	0.384	N.S.	0.611	N.S.
p/T-Akt	0.227	N.S.	63.52	<0.0001	0.003	N.S.
p/T-GSK-3β	1.771	N.S.	7.226	0.012	1.435	N.S.
p/T-p70S6K	17.00	0.0003	26.99	< 0.0001	23.75	< 0.0001
p/T-PRAS40	1.651	N.S.	107.3	< 0.0001	2.063	N.S.

**Table 2:** Two-way ANOVA summary of ethanol and NNK effects on neuronal and glial protein expression in the temporal lobe-duplex ELISA results (Immunoreactivity was measured by duplex ELISAs with results normalized to RPLPO (internal control). Results were analyzed by 2-way ANOVA and the Tukey post-hoc multiple comparisons test. F-ratios and P-values reflect independent ethanol or NNK effects, and interactive effects of ethanol and NNK. Italicized values indicate statistical trends. Results are graphed in Figures 3 and 4. See text for abbreviations.)

	Ethanol Effect		NNK Effect		Ethanol × NNK Effect	
Protein	F-Ratio	P-value	F-Ratio	P-Value	F-Ratio	P-Value
Hu	1.245	N.S.	0.885	N.S.	1.692	N.S.
MAG	7.38	0.013	47.16	< 0.0001	14.37	0.001
GFAP	1.090	N.S.	2.261	N.S.	*3.948*	0.061
ChAT	0.956	N.S.	13.24	0.0016	2.183	N.S.
AChE	0.823	N.S.	19.99	0.0002	0.688	N.S.
GAPDH	0.143	N.S.	43.58	< 0.0001	5.426	0.030
Tau	1.088	N.S.	64.95	< 0.0001	0.33	N.S.
pTau	0.842	N.S.	58.74	< 0.0001	0.922	N.S.
pTau/Tau Ratio	0.115	N.S.	0.000	N.S.	0.010	N.S.
Ubiquitin	0.0245	N.S.	32.66	< 0.0001	1.623	N.S.
4-HNE	0.232	N.S.	6.479	0.0193	5.184	0.034
ASPH-A85G6	0.003	N.S.	57.30	< 0.0001	0.177	N.S.
ASPH-A85E6	*3.34*	*0.084*	18.39	0.0004	1.525	N.S.
